# Theoretical screening of intermetallic ThMn_12_-type phases for new hard-magnetic compounds with low rare earth content

**DOI:** 10.1038/srep24686

**Published:** 2016-04-21

**Authors:** Wolfgang Körner, Georg Krugel, Christian Elsässer

**Affiliations:** 1 Fraunhofer Institute for Mechanics of Materials IWM, Wöhlerstr. 11, 79108 Freiburg, Germany; 2 University of Freiburg, Freiburg Materials Research Center (FMF), Stefan-Meier-Str. 21, 79104 Freiburg, Germany

## Abstract

We report on theoretical investigations of intermetallic phases derived from the ThMn_12_-type crystal structure. Our computational high-throughput screening (HTS) approach is extended to an estimation of the anisotropy constant K_1_, the anisotropy field H_*a*_ and the energy product (BH)_max_. The calculation of K_1_ is fast since it is based on the crystal field parameters and avoids expensive total-energy calculations with many k-points. Thus the HTS approach allows a very efficient search for hard-magnetic materials for which the magnetization M and the coercive field H_*c*_ connected to H_*a*_ represent the key quantities. Besides for NdFe_12_N which has the highest magnetization we report HTS results for several intermetallic phases based on Cerium which are interesting as alternative hard-magnetic phases because Cerium is a less ressource-critical element than Neodymium.

Due to the resource criticality of rare earth (RE) elements on the world market, especially Nd and Dy, combined with an increasing demand for high performance hard magnets for applications like wind turbines or electric vehicles, new research activities are going on worldwide in the recent years. The goal is to find new hard-magnetic compounds with comparable performance to Nd_2_Fe_14_B but with significantly fewer Nd and Dy^1^,^2^. The use of more abundant Ce instead of Nd and a significant reduction of the rare earth (RE) content relative to the transition metal (TM) content with respect to the classical Nd_2_Fe_14_B would be important steps forward in terms of cost reduction and supply reliabilty.

Recently intermetallic phases with the ThMn_12_ crystal structure have attracted a renewed experimental and theoretical interest^3^,^4^,^5^,^6^,^7^,^8^,^9^ because of the favorable composition ratio RE:TM = 1:12 and the tetragonal crystal structure which is a necessary condition for uniaxial magnetocrystalline anisotropy. A further significant increase of the magnetocrystalline anisotropy energy (MAE) can be achieved by addition of light interstitial elements (IS) like nitrogen leading to a modification of the ThMn_12_ (or 1–12) structure, denoted as ThMn_12_X (or 1–12-X) structure in the following (see Fig. 1)^6^,^7^.

The potential of phases based on the ThMn_12_X structure can be evaluated by a figure of merit, the so-called energy product (BH)_max_ which is given for some important hard-magnetic materials in Table 1. An upper bound is estimated from the magnetization M by 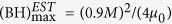 which implies the assumption that reasonably about 10% of the polycrystalline microstructure of a bulk magnet consists of nonmagnetic phases^6^. For the thoroughly studied hard-magnetic materials SmCo_5_ and Nd_2_Fe_14_B this estimation is fullfilled quite well. Hence it indicates that NdFe_12_N and related 1–12 and 1–12-X materials are indeed potentially highly interesting.

However, determining the magnetization and hence 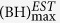 is not sufficient for the evaluation of the potential of a compound being a good hard magnet since a strong uniaxial magnetic anisotropy field H_*a*_ is required as well. H_*a*_ is connected to the anisotropy constant K_1_ and the magnetization M by the formula H_*a*_ = 2 K_1_/(*μ*_0_M). A fast and approximate determination of K_1_ is implemented in our high-throughput screening (HTS) approach to determine whether a magnetic compound has an uniaxial magnetic anisotropy or not.

For this work 1280 phases derived from the ThMn_12_ structure and the ThMn_12_X structure are investigated theoretically by a computational HTS based on an approximate but sufficiently fast and accurate density functional theory (DFT) approach^10^. After comparing the calculated key quantities M, K_1_ and H_*a*_ for well-known and important hard-magnetic materials like SmCo_5_, Sm_2_Co_17_, Nd_2_Fe_14_B, and others with experimental data we turn to the 1–12 and 1–12-X structures, respectively decorating the Th site by Ce, Nd or Sm and the Mn sites by Ti, V, Cr, Mn, Fe, Co, Ni, Cu, Zn, Al, Si or P. Furthermore, the ThMn_12_X structure containing light atoms X = B, C or N at the (2b) Wyckoff positions was screened. Besides the discussion of trends numerous RE-TM intermetallic compounds based on the ThMn_12_ like CeFe_11_TiX, (X = B, C, N), CeFe_11_Co_1_X (X = B, N) CeFe_8_Co_4_X, (X = B, C, N) or CeFe_8_Ni_4_N with high potential as low-cost hard-magnetic compounds are proposed.

Our paper is organized as follows: In section “Computational high-throughput screening” the computational HTS approach is summarized. The following section describes the determination of the anisotropy constant K_1_ which is important for the understanding of the systematic deviations between theoretical and experimental findings. In section “Validation of the HTS for known hard-magnetic compounds” the HTS approach is validated for well known hard-magnetic compounds. Possible fundamental reasons for the deviations between theory and experiment are discussed in section “Discussion of the discrepancy between calculated and experimental results”. In section “Screening results for 1–12 and 1–12-X phases” the results of the screening are presented. A concise summary concludes the paper.

## Theoretical Approach

### Computational high-throughput screening

The applied HTS approach is based the previous work of Drebov *et al.*^10^. It allows an automatized generation of new phases by substituting sets of atoms in crystal structures and then determining the formation energy, local magnetic moments and the total magnetization M. The total energies and spin polarized electronic states and densities from which all other quantities are derived are calculated with the tight-binding (TB) linear-muffin-tin-orbital (LMTO) atomic-sphere approximation (ASA) method of density functional theory (DFT) using the local spin-density approximation (LSDA)^11^,^12^,^13^,^14^,^15^. Compared to other widely employed DFT methods, the TB-LMTO-ASA method has a number of advantages for the search of new hard-magnetic materials: As an all-electron DFT method it is suitable for hard-magnetic intermetallic phases with complex crystal structures composed of transition-metal and rare-earth elements. It can treat the magnetism of localized “open-core” *f*-states interacting with delocalized valence *d*-states in a theoretically accurate and consistent manner. The minimal basis of short-ranged TB-LMTOs and the ASA for crystal potentials and electron densities together make this DFT method computationally fast and efficient at least for metallic phases with topologically close-packed crystal structures. Therefore the TB-LMTO-ASA method is a proper DFT approach for combinatorial high-throughput screening of hard-magnetic materials. More details on this computational approach are given in the following sections.

The determination of the maximum energy product (BH)_max_, the anisotropy constant K_1_ and anisotropy field H_*a*_ were added to the HTS analysis. BH_max_ is reasonably estimated by 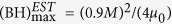 as explained in the introduction.

Further development of work of Fähnle and Hummler^15^ who implemented an evaluation at the crystal field parameters A_*nm*_ based on TB-LMTO-ASA calculations allows the determination of the anisotropy constant K_1_ and the anisotropy field H_*a*_. A short review of the single ion anisotropy approach is given in the following section.

As search criteria for hard-magnetic compounds being promising negative phase formation energies relative to the elemental sources and 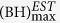 above 400 kJ/m^3^ are required. 400 kJ/m^3^ is below the already achieved 516 kJ/m^3^ of Nd_2_Fe_14_B. However, achieving such high energy products with about 50% less Nd or even without Nd using Ce would be technologically and economically very valuable. As third criterion we require that the theoretically predicted anisotropy field H_*a*_ should amount to values of at least several Tesla. All our proposed new phases have values above 10 Tesla. However, a quantitatively accurate and reliable prediction is difficult, and the reader is referred to section “Discussion of the discrepancy between calculated and experimental results” for the discussion.

### Details of the TB-LMTO-ASA calculations

The determination of the basic DFT quantities, total energy and spin dependent electron density, was performed using the framework of the LSDA. The scalar-relativistic approximation of Koelling and Harmon^29^ and the exchange-correlation functional of von Barth and Hedin^30^ in the parametrization of Moruzzi *et al.*^31^ were used.

The LSDA is valid only for weakly correlated systems which inhibits a correct treatment of the strongly localized 4*f* electrons. The problem is overcome according to Brooks *et al.*^32^ by imposing two constraints for the 4*f* charge and spin density entering the effective potential in LSDA. The constraint for the charge density fixes the number of electrons in the 4*f* core to the value of a free RE^3+^ ion. The constraint for the spin density fixes the magnetic spin moment of the 4*f* core to the value obtained from the standard Russel-Saunders coupling scheme for the free ion. In this approach, hybridization of 4*f* orbitals with other orbitals is forbidden^33^ but the radial distributions of the 4*f* charge and spin densities are calculated selfconsistently under the above mentioned constraints, is called an “open core states” approach.

In the ASA the crystal volume is subdivided into atomic spheres such that the sum of volumes of the partially overlapping spheres is equal to the total crystal volume. For the ratio of the atomic sphere radii *r* we rely on the well tested choice of *r*(RE)/*r*(TM)/*r*(IS) = 1.35/1/0.7 of previous work^34^.

Our TB-LMTO-ASA calculations included *s*, *p* and *d* orbitals (in addition to the 4*f* “open core states” as wavefunctions) and the combined-correction (cc) term^35^. For the k-point sampling of the Brillouin-zone integrals the linear tetrahedron method with Monkhorst-Pack meshes of 6 × 6 × 10 k-points was used.

### Crystal structure models

**Figure 1:**
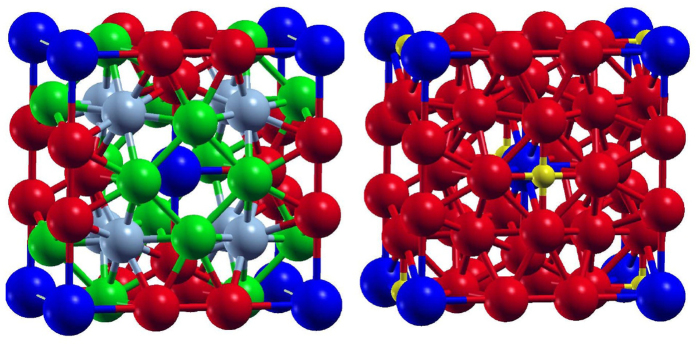
On the left a conventional unit cell model of the ThMn_12_ structure is shown. Large dark blue spheres represent RE atoms. The three different Wyckoff positions 8i, 8j and 8f occupied by TM atoms are represented by medium spheres in red, light blue and green. On the right the assumed ThMn_12_X structure with additional small yellow spheres which represent IS atoms X like B, C or N is shown.

The ThMn_12_ structure has the space group #139 (I4/mmm). For the internal structural parameters the values determined experimentally by Isnard *et al.* (see Table 4) were taken^36^. Additional interstitial atoms like B, C and N were inserted at the (2b) Wyckoff positions leading to the 1–12-X structure. The tetragonal crystal unit cells of the 1–12 and 1–12-X structure contain 26 atoms and 28 atoms, respectively. They are displayed in Fig. 1.

Tetragonal lattice constants *a* = 8.566 *Å* and *c* = 4.802 *Å* of SmFe_11_Ti^36^ were taken for our screening of all the 1–12 and 1–12-X phases.

For CeFe_11_Ti Isnard *et al.* have found that Ti occupies about one quarter of the (8i) Wyckoff positions. We assume that the Ti atoms exclusively sit on these positions for all our screening of RETM_11_Ti and related compounds.

Of course, there are variations of the lattice constants and internal parameters when different RE, TM and IS atoms are considered. However, the experimental results are somehow contradictive. On the one hand Yang *et al.*^37^,^38^ report a shortening of the lattice parameter *c* upon nitrogenation of REFe_11_TiN_*x*_ with RE = Nd or Sm. On the other hand Liao *et al.*^39^ report a slight increase of about 1%, and Akayama *et al.* have found that the *c* value increases even more^21^.

Our test calculations showed that changes of the order of 1% in structural parameters had little influence on the formation energies and magnetic moments. Beuerle *et al.*^40^ showed that taking the experimental lattice-constant data generally leads to better results for the magnetic moments than taking optimized LDA values. The influence of variations in the structural parameters on the anisotropy constant K_1_ is much bigger than few percent as can be seen by comparing work of Miyake *et al.*^7^ and Harashima *et al.*^8^. It is not clear whether experimental, LDA- or GGA-relaxed structure parameters lead to better results. We suppose that only strong changes in the lattice constants *a* and *c* due to interstitial atoms require a new relaxation of the internal structural parameters. Since for 1–12 and 1–12-X no such strong changes occur keeping all structural parameters constant only leads to small quantitative errors.

### Calculation of the magnetocrystalline anisotropy energy

For ferromagnetic crystals with uniaxial symmetry the magnetrocrystalline anisotropy energy is given in lowest order as:


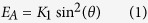


K_1_ is the first-order magnetrocrystalline anisotropy constant and has the energy-density unit J/m^3^. *θ* is the angle of the magnetization vector relative to the crystal axis of highest symmetry. Negative K_1_ values imply an easy plane whereas positive K_1_ values indicate an easy axis. For good hard-magnetic materials large positive values of several MJ/m^3^ for K_1_ are required.

For RE-TM compounds the MAE is dominated by the the RE contribution^15^. The crystal field formed by the surrounding atoms breaks the spherical symmetry for the RE atoms which implies a preferred arrangement for the RE charge. Since the spin-orbit coupling scales with the 4^t*h*^ power of the nuclear charge, Z^4^, for heavy atoms like the RE element the coupling of charge and spin degrees of freedom is important. Thus the preferred arrangement of the charge is transferred to a preferred direction of the spin leading to easy-axis or easy plane aligment of the magnetization. Clear and concise reviews of this model are given in refs 15 and 16 and in more detail in ref. 17 which show how the interaction energy of the 4*f* electrons with the surrounding electron distribution can be expanded in spherical harmonics, and that K_1_ is linear in lowest order approximation to the crystal field parameter A_20_:


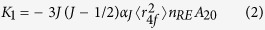


J is the total (orbital + spin) angular momentum and well defined for the 4*f* electrons. For all RE elements an ionic 3^+^ valence configuration for the 4*f* states is assumed. This is well fullfilled for most RE elements but leads to problems especially for the mixed-valence element Cerium as we will show when we discuss the numerical results. *α*_*J*_ is the so called Stevens factor^18^ which accounts for the shape of the charge distribution. The shapes of the 4*f* charge clouds are depicted for example in the book of Coey (chapter 4.4 single-ion anisotropy)^19^. 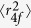 is the expectation value of the squared radius at the RE site.

Due to the localized nature of the 4*f* electrons the quantities J, *α*_*J*_ and also 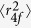 are interatomic properties quasi independent of the crystal structure and close to the values for isolated ions. Connected to the crystal structure are n_*RE*_ which is the number of RE atoms per volume and A_20_ which contains the interaction of the RE charge density *ρ*_4*f*_(*r*) with the remaining crystal charge density *ρ*(*r*):


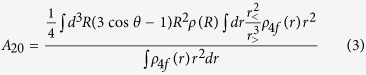


where r_<_(r ^>^) denotes the smaller (larger) of the variables r and R. These integrals have to be cut off for numerical evaluation at some radius r_*c*_. They were evaluated differently by others groups including point charge models for the large R contributions^20^. In our work we follow the approach of Fähnle *et al.*^15^ and take the sphere radius of the ASA r_*ASA*_ as cut-off radius. For RE atoms r_*ASA*_ is typically about 1.8 *Å*. Harashima *et al.*^8^ have taken a smaller cut-off radius r_*c*_ = 1.005 *Å*. In order to evaluate which cut-off works better the theoretical results are compared below to the experimentally obtained values of K_1_ and H_*a*_ of some long known and well studied hard-magnetic materials (see Table 2).

Finally, since it is relevant for the interpretation of our results, we want to note that the RE-ion anisotropy approach is only a first-order perturbation treatment. When turning the charge cloud of the 4*f* electrons away from the easy axis the reaction on the surrounding charge density in the crystal is not taken into account. A change of the 4*f* charge cloud of course modifies as well the wavefunctions (and densities) of all other electrons. This is already of second order in perturbation theory and neglected in the presented approach. A thorough critical discussion of limitations of the RE-ion anisotropy model has been given by Fähnle *et al.*^15^.

## Results and Discussion

### Validation of the HTS for known hard-magnetic compounds

In Table 2 a summary of several well studied hard-magnetic materials is given by listing data for the magnetization M, the anisotropy constant K_1_ and the anisotropy field H_*a*_.

Typically, the magnetization is well reproduced by the TB-LMTO-ASA method. The experimental and theoretical values deviate only by few percent. Only for NdFe_12_N the theoretical value clearly deviates from the experimental one. A possible reason may be that until now the NdFe_12_N phase could only be stabilized experimentally in thin films up to 360 nm thickness and not yet as a bulk material^6^.

The anisotropy represented by K_1_ or H_*a*_ is more difficult to calculate. Only numbers without digits after the decimal point are given since the approach cannot deliver data of higher significance. In Table 2 we list experimental values for K_1_ and H_*a*_ at room temperature. A comparison of the calculated values (T = 0 K) with experimental low temperature values (T ≈ 4 K) would of course be more straightforward. However, we have found less experimental data for low temperatures or extrapolated values from T = 70 K and above with an inherent uncertainty of the applied extrapolation schemes. Our intention is to identify robust general ratios between the experimental and theoretical results which allow an assessment of the screening results. Ratios of theoretical zero-temperature values to experimental room-temperature values are even more desirable with respect to permanent magnets used in practical applications.

The model calculations predict correctly the signs of K_1_ and H_*a*_ for all considered cases. Sm_2_Fe_14_B has a strongly negative K_1_ and thus no easy-axis. Y_2_Co_17_ has a vanishing K_1_ hence it has no electrons in partially filled shells. All other compounds have large positive K_1_ indicating their hard-magnetic behavior. The non-zero experimental value for Y_2_Co_17_ is due to the TM contribution to the magnetocrystalline anisotropy which is not included in the model used. The major part of the anisotropy originates from the RE atoms as explained in preceding section. The example Y_2_Co_17_ confirms that the TM contribution is rather small in RE-TM compounds^15^.

Comparing the theoretical values of K_1_ and H_*a*_ obtained with a cut-off radius r_*c*_ = r_*ASA*_ reveals an overestimation by a factor 3 to 10 of the experimental values (except for case of Ce_2_Fe_14_B). One reason for the overestimation is that at room temperature experimental K_1_ and H_*a*_ values are in general lower by a factor 2 to 3 than at zero temperature. H_*a*_ decreases from 7.0 Tesla at 4.2 K to 2.3 Tesla at 300 K for CeFe_11_Ti, e.g.^21^. For Nd_2_Fe_14_B the anisotropy field H_*a*_ decreases from 17.0 Tesla at 4 K to 7.3 Tesla at 295 K^22^. Calculating the ratio of the experimental low temperature values and the TB-LMTO-ASA values then results in percentages of 60% to 70%. In the following section we will give arguments why such an overestimation has to occur and is not a failure of the theoretical approach.

The exceptional case of Ce_2_Fe_14_B for which the experimentally found K_1_ value is only 2.8% of the calculated value is a complicated case because the valence state of Cerium in RE-TM phases is not strictly 3^+^ as assumed in our model. Capehart *et al.*^23^ found a mixed valence state of 3.44^+^ for Ce_2_Fe_14_B which implies that the theoretical model used overestimates K_1_ for Cerium containing compounds even more. In the RE-ion anisotropy model Cerium has an assumed ionic valence state of 3^+^. A valence of 4^+^ for Cerium would lead to a vanishing anisotropy. Hence the valence state depends on the RE-TM phase considered. For CeFe_11_Ti the 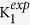/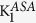 ratio is 13% and thus in the range seen also for other Nd- and Sm-containing examples. We have not found any experimental or theoretical information on the valence state of Ce in 1–12 and 1–12-X structures, respectively. Therefore the predicted K_1_ values based on the assumed valence are an upper bound for K_1_.

The values obtained with r_*c*_ = 1.005 *Å* used in ref. 8 appear to be much better at a first glance since they are in general smaller and thus closer to the experimentally determined values. However, the relative hierarchy of the theoretical anisotropy values of several examples is not as well reproduced. SmCo_5_ has about the same K_1_ value as Sm_2_Fe_17_N_3_ and Nd_2_Fe_12_N although K_1_ of SmCo_5_ should be much higher.

Our working hypothesis is that a massive overestimation of the anisotropy does not hinder a screening approach as long as the relative hierarchy is conserved and a conversion factor for the prediction of experimentally achievable values can be estimated like in the case of (BH)_max_ (Table 1). For the very well investigated and optimized phases SmCo_5_, Sm_2_Fe_17_N_3_ and Nd_2_Fe_14_B for example a factor of about 1/4 would bring 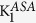 close the experimental K_1_ since the ratio 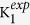/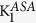 is around 25%.

For Cerium the calculated K_1_ anisotropy coefficient for the 1–12 phase CeFe_11_Ti overestimates the experimental one by about a factor of 10. For Ce_2_Fe_14_B the room temperature K_1_ is overestimated 35 times. The division of 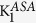 by 35 can be seen as a conservative estimate when estimating experimentally achievable values. The division of 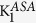 by 10 may be an optimistic estimate.

### Discussion of the discrepancy between calculated and experimental results

There is a large discrepancy between the experimental coercive field H_*c*_ of real bulk magnetic materials and the theoretical anisotropy field determined by H_*a*_ = 2K_1_/(*μ*_0_M). This is known as Brown’s paradox^24^. Its origin is the idealization of theory. Real materials are structurally, chemically and magnetically never perfectly homogeneous due to point defects and extended defects in the crystal structure, and at least equally important, surface asperities which cause strong demagnetization fields^19^,^25^. The reversal of magnetization is strongly connected to small nucleation or pinning centers in the material. Our anisotropy model described above is not containing any inhomogenities or surface effects. Furthermore, in theory it is assumed implicitly that magnetization is rotated in a coherent mode which means that magnetization remains uniform everywhere and rotates unison.

Real magnetic single crystals are never free of point defects, have surfaces or interfaces, and are not in perfect single-domain configurations like assumed in the model calculations. This implies that experimentally determined K_1_ values for single crystals are very likely lower than the theoretically determined values. In experiments extremely high fields would be necessary to obtain completely saturated magnetic single-domain single crystals. For example in Fig. 1 of ref. 26 one can see that K_1_ differs with the applied external field. But even in an extremely high external field a magnet of one pure phase contains point defects, dislocations and little surface asperities which can counteract to the single domain state. Thus the critical threshold for rotating the magnetization is lower than for an ideal magnetic crystal.

We think that the main reason for the discrepancy is that the charge distribution of all electrons needs to be determined selfconsistently for the parallel and perpendicular arrangment. As mentioned before in the RE-ion anisotropy approach only the first-order perturbation is taken into account which is the change of the energy when rotating the charge cloud of the 4*f* electrons with respect to the surrounding charge cloud of all other electrons. The reaction on the charge density, i.e. a modification of the wavefunctions (second order perturbation) is neglected. Selfconsistent MAE calculations based on differences of total energies (for the magnetization parallel and perpendicular, respectively, to the easy axis of the crystal) take this reaction into account. This and the inclusion of the TM contribution to the MAE are clearly advantages of the total-energy calculations. However, in order to obtain quantitatively accurate values for K_1_ and H_*a*_ dense k-meshes of several thousands of k-points are needed for the Brillouin-zone integrals^27^,^28^. This make total-energy calculations for HTS rather disadvantageous.

### Screening results for 1–12 and 1–12-X phases

In Table 3 a selection of the 1280 investigated phases are listed. The upper part contains a systematic collection of phases which allows to see trends when changing from 1–12 to 1–12-X, from REFe_12_ to REFe_11_Ti, or exchanging the interstitials B, C and N. The lower part of the table proposes some further promising compounds.

#### Trends

Since the TB-LMTO-ASA approach is able to calculate the magnetization M accurately and reliably (see Table 2), we interpret discrepancies between theory and experiment as a hint to further optimization potential. NdFe_12_N was successfully synthesized in thin films with a reported magnetization of 1.66 T^6^. Our value of 2.06 T indicates an upper bound for a bulk material. From the magnetization M the energy products 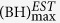 in the first column of Table 3 are derived according to 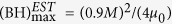.

Concerning the anisotropy we find that Ce and Nd, both having oblate 4*f* charge clouds, lead to easy-axis magnets for all named 1–12 and 1–12-X phases whereas Sm with its prolate 4*f* charge cloud always leads to easy-plane magnets. These results are partially at variance to the work of Harashima *et al.* that report a positive K_1_ for SmFe_12_. Also they predict a negative K_1_ for NdFe_12_ which contradicts our prediction of an easy axis. Taking exactly their structural parameters and redoing the K_1_ calculation did not change the sign for our results but has led to values closer to zero. Probably one can say that these K_1_ values are very small anyway and should be considered to be approximately zero, and differences in sign are thus uncritical.

A clear trend which was already found and discussed by Miyake *et al.*^7^ is the strong increase in K_1_ when going from 1–12 to 1–12-X phases. At least for the doping with nitrogen this was already observed in experiments^6^,^21^. According to our model calculations the increase can be about one order of magnitude irrespective of the interstitial element used. Doping with B always leads to smaller values than doping with C or N. As a general argument we assume that B changes least the charge distribution around the RE atoms and thus has the lowest impact on the RE anisotropy.

Going from REFe_12_X phases to REFe_11_TiX phases clearly reduces the magnetization and increases the anisotropy. The above mentioned results for NdFe_12_N and NdFe_11_TiN are apparently in good agreement with experimental findings^6^. Already going from REFe_12_ to REFe_11_Ti reduces the magnetization and increases the anisotropy. But REFe_12_ compounds are experimentally unstable^6^, as reflected in high formation energies in the calculations.

For NdFe_11_TiX (X = B, C, N) our results deviate from the theoretical findings of Harashima *et al.*^9^. Besides the chemical change they take the structural change by individual relaxations of the respective structures into account. Experiments will show whether such big differences in the anisotropy between the NdFe_11_TiX phases (X = B, C, N) exist or whether the change is rather comparable, similar as the change seen when going from Nd_2_Fe_14_B to Nd_2_Fe_14_C (see Table 2).

Substituting Ti on the 8i positions by V, Cr, Cu, Zn, Al, Si, or P has not lead to promising compounds. In general this formation energies are increased making their existence less likely, and the magnetizations and anisotropies are lower than for Ti containing compounds. Therefore none of these screened compounds entered Table 3.

#### Promising Compounds

From the upper part of Table 3 NdFe_12_X, NdFe_11_TiX, CeFe_12_X and CeFe_11_TiX (X = B, C, N) provide good key data. Nd containing compounds achieve higher magnetizations relative to Ce containing compounds due to the local magnetic moments of Nd atoms. Also we expect the anisotropy for Nd to be higher than for Ce. A division of the theoretical 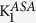 and 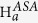 values of Nd compounds by about 4 should indicate an upper bound for the experimentally achievable values (see discussion of Table 2). For Ce_2_Fe_14_B only about 3% of the values determined by TB-LMTO-ASA is achieved, so a division by 35 of the 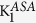 and 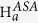 values Cerium compounds is a conservative estimate for the potential (cf. discussion in Section 2). However, CeFe_11_Ti achieved more than 10% of the idealized 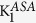 and 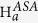 values. Thus in case that the mixed valence of Cerium in 1–12 and 1–12-X phases is less dominant than in 2-14-1 phases the model assumptions work better and the overestimation by theory is lower.

The substitution of Mn on Fe positions in the 1–12 and 1–12-X structures also did not give any promising compounds since the magnetization decreased in most cases significantly (cf. ref. 10 for 1-14-2 phases). However, the partial substitution of Co or Ni for Fe leads to quaternary phases like CeFe_11_Co_1_X (X = B, N), CeFe_8_Co_4_X (X = B, C, N) or CeFe_8_Ni_4_N with rather high hard-magnetic potentials. They are expected to have anisotropy fields of about 20 Tesla and higher. Although, this is only half of the anisotropy field of SmCo_5_, but in combination with much higher magnetizations much higher energy products should be achievable. Furthermore, the expensive Sm would be replaced by the uncritical Ce, and the Co may lead to rather high Curie temperatures.

### Summary

We have studied the magnetic properties of 1–12 and 1–12-X phase on the search for promising hard-magnetic phases by HTS calculations based on a fast TB-LMTO-ASA approach which predicts the magnetization in good accuracy. This allows an estimation of the maximum energy product (BH)_max_ which is an important figure of merit for permanent magnets. We determined the anisotropy for the single domain state of the perfect single crystal. Although our values overestimate the experimentally obtained values of well-known magnets it was shown that at least a trend of the anisotropy constant K_1_ and the anisotropy field H_*a*_ can be reproduced. Thus by scaling down the theoretical result by a factor 4 for Nd and Sm and a factor 10 to 35 for Cerium leads to estimates for upper bounds of K_1_ and H_*a*_ that are achievable in experiments. Our screening of 1280 phases has lead to several promising phases like NdFe_12_X or NdFe_11_TiX (X = B, C, N) with energy products (BH)_max_ up to 600 kJ/m^3^ and anisotropy fields up to 10 Tesla. Ce containing compounds like CeFe_11_TiX, (X = B, C, N), CeFe_11_Co_1_X (X = B, N) CeFe_8_Co_4_X (X = B, C, N), or CeFe_8_Ni_4_N have lower energy products but have the advantage of being less resource-critical due to the avoidance of Nd.

We recommend the above proposed RE-TM-IS compounds as worth and promising to be investigated further experimentally.

## Additional Information

**How to cite this article**: Körner, W. *et al.* Theoretical screening of intermetallic ThMn_12_-type phases for new hard-magnetic compounds with low rare earth content. *Sci. Rep.* **6**, 24686; doi: 10.1038/srep24686 (2016).

## Figures and Tables

**Table 1:** Comparison of the estimtated (BH)_max_^EST^ calculated from the magnetization M obtained with TB-LMTO-ASA and the experimentally achieved 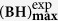 given in [kJ/m^3^].

System	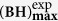 [kJ/m^3^]	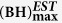 [kJ/m^3^]
SmCo_5_	219^a^	174
Sm_2_Co_17_	336^a^	240
Sm_2_Fe_17_N_3_	472^a^	459
Nd_2_Fe_14_B	516^a^	563
NdFe_12_N	*	686

^*a*^ref. 41, *only thin film but no bulk phase synthesized so far.

**Table 2:** Comparison of the magnetization, the anisotropy constant K_1_ and the anisotropy field H_*a*_ calculated with TB-LMTO-ASA with experimental data taken from ^*a*^ref. 19 (Table 11.1), ^*b*^ref. 22, ^*c*^ref. 42, ^*d*^ref. 6 (no bulk but films of thickness up to 360 nm) and ^*e*^ref. 21.

System	*μ*_0_M^exp^ [T]	*μ*_0_M^*ASA*^ [T]	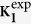 [MJ/m^3^]	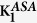 [MJ/m^3^]	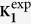 / 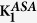	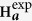 [T]	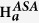 [T]	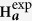 / 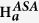
SmCo_5_	1.07^*a*^	1.04	17.2^*a*^	69 (26)	25%	40.4^*a*^	166	25%
Sm_2_Co_17_	1.25^*a*^	1.22	4.2^*a*^	25 (11)	17%	8.4^*a*^	53	16%
Y_2_Co_17_	1.26^*a*^	1.17	−0.34^*a*^	0 (0)		−0.7^*a*^	0	
Sm_2_Fe_17_N_3_	1.54^*a*^	1.69	8.6^*a*^	27 (25)	32%	14.0^*a*^	40	35%
Ce_2_Fe_14_B	1.44^*b*^	1.75	1.5^*b*^	54 (14)	2.8%	2.6^*b*^	76	3.4%
Nd_2_Fe_14_B	1.86^*b*^	1.87	4.9^*a*^	19 (6)	26%	6.6^*a*^ 7.3	26	25%^*a*^
Nd_2_Fe_14_C	1.61^*b*^	1.85	4^*c*^	24 (7)	17%	6.3^*c*^	32	20%
Sm_2_Fe_14_B	1.65^*b*^	1.62	≤ −13^*a*^	−31 (−10)	42%	≥15^*b*^	−47	32%
NdFe_12_N	1.66^*d*^	2.06	5.3^*d*^	47 (28)	11%	8*d*	57	14%
NdFe_11_Ti	1.70^*e*^	1.65	1.35	4 (2)	31%	2.0^*e*^	7	28%
CeFe_11_Ti	1.55^*e*^	1.57	1.4^*e*^	11 (4)	13%	2.3^*e*^	18	13%

The experimental values for K_1_ and H_*a*_ in columns 3 and 5 are given for room-temperature. Experimental magnetization values are given for T ≈ 4K. The calculated K_1_ values in brackets are determined with a cut-off radius r_*c*_ = 1.005 *Å* as in ref. 8.

**Table 3:** Selection of HTS results: Key quantities like the estimated energy products 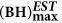, the magnetization M, the anisotropy constant K_1_ and the anisotropy field H_*a*_ calculated with TB-LMTO-ASA are listed together with experimental data taken from ^*a*^ref. 21, ^*b*^ref. 22, ^*c*^ref. 5, ^*d*^ref. 6, ^*e*^ref. 8, ^*f*^ref. 9.

System	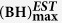 [kJ/m^3^]	*μ*_0_M^*ASA*^ [T]	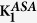 [MJ/m^3^]	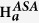 [T]	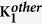 [MJ/m^3^]	*μ*_0_M^*exp*^ [T]	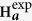 [T]
NdFe_12_*	636	1.99	3	3	−2.2^*e*^		
NdFe_12_B	611	1.95	45	58			
NdFe_12_C	617	1.96	47	60			
NdFe_12_N	686	2.06	47	57	9.91^*e*^	1.66^*d*^	8^*d*^
NdFe_11_Ti	438	1.65	4	7	−0.58^*e*^ 1.70^*a*^	1.70^*a*^	2.0^*a*^
NdFe_11_TiB	432	1.64	48	72	−0.70^*f*^		
NdFe_11_TiC	432	1.64	50	76	2.6 ^*f*^		
NdFe_11_TiN	487	1.74	49	71	11.3^*e*^ 10.6^*f*^	1.48^*d*^	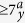
CeFe_12_*	586	1.91	4	5			
CeFe_12_B/C/N	556/568/630	1.86/1.88/1.98	127/137/139	170/182/175			
CeFe_11_Ti	396	1.57	11	18		1.19^*c*^ 1.55^*a*^	2.96^*c*^ 2.3^*a*^
CeFe_11_TiB/C/N	396/391/443	1.57/1.56/1.66	134/145/148	213/232/222			
SmFe_12_*	538	1.83	−5	−6	2.4^*e*^		
SmFe_11_Ti	357	1.49	−8	−13	−0.52^*e*^		
SmFe_11_TiN	401	1.58	−73	−115	−20.4^*e*^		
SmFe_12_N	580	1.90	−71	−93	−18.1^*e*^		
CeFe_11_Co_1_B/N	536/605	1.83/1.91	129/142	176/183			
CeFe_8_Co_4_B/C/N	464/464/521	1.70/1.70/1.80	116/141/146	168/208/203			
CeFe_8_Ni_4_N	417	1.61	167	260			
NdFe_11_Co_1_B/C/N	586/586/661	1.91/1.91/2.03	46/48/48	60/63/59			
NdFe_8_Co_4_B/C/N	520/505/574	1.80/1.77/1.89	41/49/50	57/69/67			

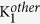
 denotes theoretical results from other groups. A star (^*^) indicates phases with high formation energies and thus instability. _y_: in this experiment the exact composition was NdFe_11_TiN_1,5_^21^.

**Table 4:** Internal structural parameters used for the calculations.

Wyckhoff position	X	Y	Z
RE (2a)	0	0	0
TM (8i)	0.3534	0	0
TM (8j)	0.2753	0	0
TM (8f)	0.25	0.25	0.25
IS (2b)	0	0	0.5

The values for (8i), (8j) and (8f) were obtained by Isnard *et al.*^36^ from neutron powder diffraction.
